# Resulting Sequelae of an Immunosuppressive State in a Pediatric Patient Status Post Bone Marrow Transplant for the Curative Management of Myelodysplastic Syndrome

**DOI:** 10.7759/cureus.83318

**Published:** 2025-05-01

**Authors:** Emily D'Arpa, Margaret Honeycutt, Harsha Bhagtani

**Affiliations:** 1 Department of Pediatrics, Edward Via College of Osteopathic Medicine, Blacksburg, USA; 2 Pediatrics, Clinch Valley Physician Practices, Cedar Bluff, VA, USA

**Keywords:** graft versus host, hypogammaglobulinemia, immunodeficiency, myelodysplastic syndrome, pediatric, transplant

## Abstract

Myelodysplastic syndrome is a clonal disorder of hematopoietic stem cells leading to dysplasia and ineffective hematopoiesis. The typical presentation is asymptomatic with incidental lab findings consistent with varying degrees of pancytopenia. This syndrome is commonly diagnosed in older individuals and tends to result from chemical and/or radiation exposures; however, this patient presents as a unique manifestation of myelodysplastic syndrome in a six-month-old male. For curative management, the patient had an initial haploidentical hematopoietic stem cell transplant, followed by another, due to early graft rejection.

In the following years leading to the present time, the patient has had complications due to a persistent immunodeficient state. He has been evaluated and managed for seizures, anemia, chemotherapy-induced cardiomyopathy, hypogammaglobinemia, failure to thrive, recurrent infections, graft versus host disease, malnutrition, and hypertransaminasemia with hepatosplenomegaly. He has had resolution of his cardiomyopathy and seizures but continues to manage his persistent immunodeficiencies, hepatomegaly, and slow weight gain. It is hard to discern if the multitude of dysfunctions arises from pharmacologically induced immunodeficiency, from parental and environmental neglect, if the manifestations compounded one another, or if the above factors combined to create the unique constellation of clinical manifestations.

This patient provides insight into the complexities associated with understanding unique manifestations in dysplastic and oncological conditions, as well as managing complications arising from treatment. This case raises awareness of medical complications in the context of parental neglect and the importance of managing them both. The authors received consent from the patient’s guardian to use their data for this report.

## Introduction

Myelodysplastic syndrome (MDS) is a clonal disorder of hematopoietic stem cells characterized by ineffective hematopoiesis and associated dysplasia. The primary manifestation of MDS tends to be asymptomatic cytopenia: anemia, leukopenia, thrombocytopenia, or any combination of the three [1]. MDS is commonly diagnosed in older patients, with the most affected demographic being white males older than 80 years, with an occurrence of 4.9 per 100,000 persons. Pediatric MDS has a much lower incidence of about one to four cases per million persons per year and does not have a predilection for either males or females [2]. Clinical and morphological differences between adult and pediatric MDS have allowed for pediatric MDS to be described as a distinct entity [3]. One differentiating factor lies in the etiology. Whereas most adult MDS is associated with environmental and iatrogenic exposures, childhood MDS is frequently linked to underlying genetic defects such as chromosomal abnormalities.

Since pediatric patients tend to lack the exposure profile typical of adult MDS patients, it is important to perform a karyotype analysis for the potential of identifying an underlying chromosomal abnormality. Karyotyping is performed to give physicians a baseline level of understanding regarding the progression of a disease state through an analysis of chromosomes. The karyotype can reveal if there are abnormalities in the number, shape, and size of chromosomes, and furthermore, the abnormalities can be classified as either numerical or structural. Numerical refers to aneuploidies with either missing or extra chromosomes, and structural means translocations, duplications, and rearrangements [4]. As stated earlier, chromosomal abnormalities leading to a complex karyotype are more common in pediatric rather than adult cases of MDS. To further the differential, most patients with MDS have numerical anomalies, whereas acute myeloid leukemia (AML) patients tend to have structural abnormalities [2].

The minimal unique symptomology of MDS, in addition to a lack of consensus in diagnosing MDS in children, leads to a common misdiagnosis of refractory anemia. There are no single, specific histopathological features that define MDS; diagnosis instead rests on findings from both peripheral blood and bone marrow samples [1]. Diagnostic criteria require at least one peripheral blood cytopenia, blasts accounting for less than 20% of bone marrow cells, and evidence of dysplasia greater than 10% of cell lines (red cell precursors, granulocytes, and/or megakaryocytes). Specifically for pediatric patients, the World Health Organization (WHO) has defined and since revised pediatric characteristics that help to classify varying myeloproliferative disorders in pediatric patients while also providing a prognosis based on the classification. The 2008 WHO criteria separated myeloproliferative disorders into three groups: MDS, juvenile myelomonocytic leukemia, and Down syndrome disorders, and then further classified refractory cytopenia, refractory anemia with excess blasts, and refractory anemia with excess blasts in transformation [2]. The majority of pediatric MDS cases fall into the subdivision of refractory cytopenia. Once the blasts extend to greater than 20%, there is a greater concern that the initial MDS has progressed to a leukemic state and no longer qualifies for a diagnosis of MDS: an elevated percentage of blasts correlates with a poorer prognosis. Flow cytometry is not very specific for the diagnosis of MDS; however, it may be performed to assist in diagnosis, especially if bone marrow findings are suboptimal or equivocal. Other helpful factors in the diagnostic process are the use of immunostains; positive staining of immunostains such as myeloperoxidase (MPO), CD34, CD117, CD61, CD68, CD20, and/or CD3 may be indicative of MDS and should be considered for more thorough workup.

One of the concerning factors linked to the prognosis of MDS is the risk of it evolving into a leukemic state. MDS typically presents asymptomatically, and if it presents with only refractory cytopenia, it may be permitted to silently progress to undiagnosed leukemia. The progression may occur rapidly over a few months, or it may take years; this is dependent on prognostic factors classifying the MDS as more severe or not. To prevent the possibility of progression, it is of utmost importance to accurately diagnose and treat MDS.

MDS demonstrates nonresponse to cytotoxic chemotherapeutic agents, but some success has been shown with hypomethylating agents [1]. Bone marrow transplant (BMT) is the only curative option currently available to treat childhood MDS, making it the first-line treatment of choice in children [2]. BMT is utilized to help generate functional stem cells to replace dysfunctional ones, as well as supplement the function of currently present bone marrow [5]. Prior to performing BMT, human leukocyte antigen (HLA) typing is performed to determine the most suitable donor. HLAs are blood proteins that reside on the cellular surface and function predominantly in alloimmunity by recognizing what is self and what is foreign, unrecognized material. If foreign material is recognized, HLAs begin the process of mounting an immune response. The best-suited candidates for stem cell donation are individuals who are HLA-matched and related donors; however, finding a perfectly matched donor is not always possible and may take a long time. Haploidentical donors are HLA-mismatched first-degree relatives. Though this helps since patients have more rapid access to a donor, there is an increased risk of developing graft versus host disease (GVHD). GVHD occurs when the transplanted immune cells attack the recipient of the transplant. In patients receiving an allogenic stem cell transplant, the risk of developing GVHD is high: up to 50% of patients [6].

This is a high risk of complications from a curative treatment in which benefits outweigh the costs; however, there have been attempts to reduce the number of affected post-transplant patients. A study followed 72 transplant recipients who received a haploidentical donor’s hematopoietic progenitor cell in addition to NK cell transplant (HAPNK1) with a secondary goal of estimating the incidence and severity of GVHD [7]. The addition of the NK cells helps enhance engraftment to prevent graft rejection, and their innate cytotoxic abilities to lyse alloreactive T cells from the donor induce the GVHD response. The regimen consisted of a preparative regimen of total lymphoid irradiation (TLI) and granulocyte colony-stimulating factor (G-CSF), alongside the following chemotherapeutics and immunosuppressants: fludarabine, cyclophosphamide, thiotepa, and melphalan, to then be followed by an infusion of the donor cells. After monitoring for one year, 22.2% of patients were found to have chronic GVHD. In addition to GVHD, patients following HPSCT have other complications to overcome, which may occur because of the attempts to decrease the risk of graft rejection: patients undergo immunosuppressive therapies, which can then precipitate delayed immune reconstitution and lead to increased risk for infection.

The current case presents a young pediatric patient with minimal somatic complaints. His clinical workup led to a diagnosis of MDS, which emphasizes the importance of considering a broad differential and pursuing a thorough workup. His diagnosis and treatment resulted in a slew of complications, which will be further discussed in this report.

## Case presentation

The patient was a nine-year-old Caucasian male status post two haploidentical hematopoietic stem cell transplants (haplo-HSCT) for a history of myelodysplastic syndrome and left orbital chloroma diagnosed on 3/20/2016 at six months of age. The patient has been in clinical remission for years, followed closely by his hematology/oncology team. As a result of his chemotherapy, radiation, and immunosuppressed state for his haplo-HSCT, the patient has had a resulting constellation of complications: failure to thrive, acquired hypogammaglobinemia, persistent anemia, GVHD, pulmonary dysfunction, chemotherapy-induced cardiomyopathy, hepatomegaly with hypertransaminasemia, and growth hormone resistance. In addition to his medical presentations, the patient’s case has the additional complicating factor of environmental neglect by his guardians.

The onset of symptoms related to the diagnosis of his MDS began when the patient’s parents noticed dermatological abnormalities. These skin changes had been present since birth but began increasing in size and the number of areas affected. The parents subjectively reported a purple-blue rash localized on their son’s scalp that, around six months of age, had noticeably spread onto his back and chest. On exam, the lesions were all slightly raised with larger areas on the scalp and more diffuse spread to the ones on his back and sides. A skin biopsy was obtained, and it was defined as extramedullary hematopoiesis; further workup was pursued.

A review of his birth history reveals he was born at 38 weeks via C-section; the patient was macrosomic at 11 pounds secondary to maternal gestational diabetes. He did have an extended stay at the hospital in the NICU for 11 days due to cardiomegaly and poor lung function. He was cleared by cardiology at that time. The patient was stabilized and allowed to go home. He had no family history of autoimmune problems or constitutional growth delay. Otherwise, the patient’s medical history up to this time was unremarkable.

His initial workup is demonstrated in Table 1. Laboratory findings revealed a low absolute neutrophil count in the setting of a viral upper respiratory infection. Other abnormalities included low mean corpuscular volume (MCV), low mean corpuscular hemoglobin, and an elevated red cell distribution (RDW), which could indicate iron deficiency or microcytic anemia. X-ray imaging was also performed for potential underlying bone pathology in areas corresponding to the skin lesions, with no evidence of acute abnormalities.

**Table 1:** Initial laboratory workup: complete blood count with differential WBC: White blood cells, RBC: Red blood cells, MCV: Mean corpuscular volume, MCH: Mean corpuscular hemoglobin, RDW: Red cell distribution width, MPV: Mean platelet volume

	Patient’s Value	Reference Value
WBC	8.74	6.40 – 13.00 k/uL
RBC	3.92	3.00 - 5.40 k/uL
Hemoglobin	10.4	10.0 – 18.0 g/dL
Hematocrit	32.0	31.0 – 55.0%
MCV	81.6	83.0 – 95.0 fL
MCH	26.5	28.0 – 32.0 pg
RDW	16.8	11.0 – 14.0%
MPV	11.4	9.0 – 12.0 fL
Platelets	180	150 – 450 k/uL
Lymphocytes Absolute Count	6.94	3.4 – 9.00 k/uL
Monocyte Absolute Count	0.34	0.50 – 1.10 k/uL
Eosinophil Absolute Count	0.17	0.00 – 1.00 k/uL
Basophil Absolute Count	0.00	0.00 – 0.20 k/uL
Neutrophils Absolute Count	0.86	1.00 – 8.50 k/UL

A skin punch biopsy was acquired from one of the lesions over his lumbar region, which was suggestive of leukemic infiltrate with a majority of immature myeloid differentiation. The immunostaining results are presented in Table 2. 

**Table 2:** Skin biopsy immunostaining results MPO: Myeloperoxidase

Positive	MPO, CD43
Negative	CD56, CD117, CD34, CD68, and CD163

The positive immunostaining, as well as persistent neutropenia, led to a consideration of a diagnosis of aleukemic leukemia cutis. Cytogenetics revealed a complex karyotype (46, XY, add(4)(q36), del(4)(q25q35),-10,-11,+2mars(18)/46, XY(2)). He demonstrated both structural and numerical anomalies: chromosome 4 yielded structural changes and chromosomes 10 and 11 both had numerical changes via loss. Two marker chromosomes likely resulted from a complex rearrangement between chromosomes 10 and 11. Fluorescence in situ hybridization (FISH) was also performed using an AML panel, and it yielded normal hybridization patterns.

Bone marrow evaluation revealed normocellular bone marrow with trilineage hematopoiesis and a large, atypical population of left-shift myeloid cells (promyelocytes). The results of the bone marrow aspirate are found in Table 3. Promyelocytes were elevated along with an elevated myeloid-to-erythroid ratio. The patient had low-band neutrophils, segmented neutrophils, and lymphocytes present. No Auer rods or ringed sideroblasts were present. The peripheral blood smear revealed normochromic normocytic anemia with occasional nucleated red blood cells and the presence of blasts. While obtaining the sample for bone marrow studies, a repeat skin biopsy was obtained from the upper back. The findings were consistent with the original biopsy of myeloid infiltrate with a marked left shift that stained positive for MPO, Muramidase (lysozyme), and Leder. These findings, paired with the clinical presentation, were representative of the diagnosis of myelodysplastic syndrome due to the lack of involvement of dysplastic cells in the blood and bone marrow, limited only to the periphery, paired with the presence of a small percentage of blasts.

**Table 3:** Bone marrow aspirate findings

	Patient’s Value	Reference Value
Blasts	1	1 – 4%
Promyelocytes	40	0 -2%
Myelocytes	16	1 – 5%
Metamyelocytes	10	7 – 15%
Band neutrophils	4	9 – 19%
Segmented neutrophils	7	22 – 36%
Eosinophils	2	1 – 3%
Basophils	1	0 – 1%
Monocytes	2	0 – 2%
Lymphocytes	6	38 – 56%
Myeloid:Erythroid Ratio	7.5:1	1:1 – 4:1 ratio

Once the diagnosis of MDS was made, the patient was started on non-protocol single-agent azacitidine, a hypomethylating chemotherapeutic. He remained on this single-agent therapy for five months, when he was then transitioned to induction chemotherapy and HAPNK1 conditioning in preparation for his haplo-HSCT. The patient’s donor was his father; both patient and donor were O-positive, but due to an incomplete HLA match, the plan was to proceed with the haplo-HSCT while attempting to minimize the risk of transplant complications. He received TLI one week prior to the transplant. His first transplant was on 11/22/2016, for which he received the following chemotherapeutics: fludarabine, cyclophosphamide, thiotepa, and melphalan, alongside immunosuppressants (maraviroc and mycophenolate mofetil). One week after the transplant, he received the NK cell transplant, which was the standard treatment for HAPNK1. Despite efforts to minimize graft rejection, the patient developed an acute immunologic response and mixed chimerism. Mixed chimerism occurs when the lymphohematopoietic system of the recipient of a transplant has a combination of both recipient (host) and donor cells. Early manifestations of mixed chimerism may be predictive of graft rejection. He then underwent a salvage transplant one month after the initial transplant (12/28), with a CD3-depleted graft following total body irradiation (TBI). Reduced-intensity conditioning via fludarabine, cyclophosphamide, rabbit anti-thymocyte globulin (rATG), and rituximab, alongside mycophenolate mofetil, was utilized, followed by a low-dose memory donor lymphocyte (CD45RA-depleted DLI) replacement. This graft placed the patient in a T-lymphopenic state, therefore leaving him in a state of delayed immune recovery; however, it has since been proven to be curative of this patient’s MDS. He has since been found to be in clinical remission.

As a result of his immunodeficient state and later determined environmental neglect, the patient has also been followed by physicians from varying specialties, including endocrinology, gastroenterology, cardiology, neurology, and pulmonology, in addition to hematology/oncology and primary care. He is monitored closely for all clinical manifestations that have occurred as a result of his immunodeficient state and his environmental neglect.

For example, at the time of his diagnosis of MDS, the patient was found with microcytic anemia since he had persistent anemia. Blood smears repeatedly reveal moderate microcytic hypochromic anemia with a high RDW. Iron studies showed low iron, elevated ferritin, and a normal total iron binding capacity (TIBC) as demonstrated in Table 4. His anemia is managed with NovaFerrum® pediatric iron supplement and attempts to increase diet variety, as he prefers to consume mainly cow’s milk, which may contribute to his persistent anemia.

**Table 4:** Iron study TIBC: Total iron binding capacity

	Patient’s Value	Reference Value
Iron	12	50 – 120 mcg/dL
TIBC	359	169 – 477 mcg/dL
Iron Saturation	3.3	20 – 50%
Ferritin	227	7 – 140 ng/mL

Early in the post-HCT period, the patient had what were described as “breath-holding spells” that led to seizure-like movements. The patient was managed with levetiracetam until he was three years old and followed up with neurology. An electroencephalogram (EEG) revealed an encephalopathic process. He has since been taken off the antiepileptics and remains seizure-free. He additionally follows up with neurology for monitoring following the TBI he received via magnetic resonance imaging (MRI). His most recent brain MRI was obtained on 9/29/2024 and revealed no acute intracranial processes, but did demonstrate persistent white matter disease. The primary differential is consistent with posttreatment leukoencephalopathy that may manifest as difficulties in school and cognitive impairment. 

The patient was initially found to have chemotherapy-induced cardiomyopathy. He was on multiple chemotherapeutic agents and continued to receive different therapies up until three years of age, which likely plays a contributing role. This cardiomyopathy was managed with metoprolol until a normal echocardiogram (ECHO) in 2020. The most recent electrocardiogram and ECHO revealed sinus rhythm, normal systolic and diastolic function, normal chamber size, and a preserved ejection fraction at 65-70%. He continues to follow up with cardiology without new concerns, no activity restrictions, or the need for subacute bacterial endocarditis (SBE) prophylaxis.

Allogenic HSCT in children commonly results in hypogammaglobulinemia; this patient had no detectable immunoglobulin (Ig)M or IgA levels, and both have maintained a persistent low state as demonstrated in Table 5. He was started on intravenous immunoglobulin (IVIG) infusions in 2020. After six months of IVIG, he was transitioned to a subcutaneous immunoglobulin therapy (Hizentra 20%, CSL Behring LLC, Pennsylvania, US), which he receives weekly. The Hizentra serves as replacement therapy rather than a permanent treatment, so he will remain on Hizentra for the remainder of his life. This patient’s hypogammaglobulinemia manifested as frequent infections. He has had recurrent otitis media leading to bilateral tympanostomy tube placement, sinusitis, upper respiratory tract infections, and pneumonia.

**Table 5:** Immunoglobulin levels: initial and most recent values

	Patient’s Value (1/12/19)	Patient’s Value (9/26/23)	Reference Value
IgA	< 4	< 40	19 – 220 mg/dL
IgG	574	-	341 – 1960 mg/dL
IgM	< 1	< 2	43 – 163 mg/dL

Per growth charts and parental reports, he was growing and developing normally until the stem cell transplant. There is no family history of constitutional growth delay or autoimmune problems. His mother’s family is taller than his father’s, but there was no concern for familial short stature. The patient’s most recent bone age exam was performed when he was eight years seven months, and his bone age was approximated at six years, confirming delayed bone age. This finding does not place him on track to reach his mid-parental height, which is close to the 75th percentile.

The earliest observation of his delayed growth was two years after his transplants: his weight had remained unchanged from his follow-up one year prior, while his height increased by 7 cm. His body measurements most recently are as follows: he is less than the first percentile in both weight and stature for age (18.5 kg and 113.5 cm, respectively), his body mass index (BMI) for age is the sixteenth percentile (14.64 kg/m2), and his mid-parental height is the thirty-seventh percentile (1.741 m). Endocrinology has noted a preserved BMI with an abnormally slow growth velocity at 1.9 cm/year, which places him below the third percentile. He has no vitamin D abnormalities. His laboratory results are shown in Tables 6-7, revealing low insulin-like growth factor (IGF) binding protein 3 and normal GH production. His peak GH production was in the afternoon and was greater than the upper limit of normal required for GH deficiency. Although GH appears to be made normally, alongside the low IGF binding protein 3 and clinical short stature, it is likely that the patient has GH resistance. He was started on Genotropin 12 mg/mL as a GH replacement and continues to follow up with endocrinology.

**Table 6:** Endocrinology laboratory results ACTH: Adrenocorticotropic hormone, IGF-1: Insulin-like growth factor 1, TSH: Thyroid-stimulating hormone

	Patient’s Value	Reference Value
ACTH	13.8	7.2 – 63.3 pg/mL
AM Cortisol	11.5	1.5 – 12.3 ug/dL
IGF-1	45	20 – 347 ng/mL
IGF-1 Z-score	-1.5	Between -2.0 and + 2.0
IGF binding protein 3	1500	1932 – 5858 ng/mL
Free T4 (Thyroxine)	1.6	0.8 – 1.3 ng/dL
TSH	1.72	0.86 – 5.70 uIU/mL
Hemoglobin A1C	5.5	4.6 – 5.6%

**Table 7:** Growth hormone stimulation test GH: Growth hormone

Time	Cortisol Patient Value	Cortisol Reference Value	GH	GH Reference Value
8:15	11.2	1.5 – 12.3 ug/dL	0.11	> 10 ng/mL
10:15	-	-	4.35	-
10:41	-	-	14.10	-
11:15	-	-	7.94	-
11:45	12.4	-	2.21	-
12:45	-	-	5.44	-
13:15	-	-	17.80	-

From both his chemotherapy and TBI, there were known risks that he may develop endocrinological complications such as hypopituitarism, thyroid nodules, metabolic syndrome, and delayed skeletal development. However, this patient additionally suffered from parental neglect, which likely compounded his malnutrition state and contributed to his failure to thrive (FTT). He ultimately required a hospital admission to the pediatric intensive care unit (PICU) for electrolyte imbalances, dehydration, and malnourishment in September of 2023 due to being lost to follow-up with his oncology center, failing to return for laboratory testing, and being noncompliant with his treatments. He was brought in by Child Protective Services (CPS) after an anonymous person reported he was sick and not receiving adequate care. Objective findings were pallor with abdominal distension, microcytic anemia, hypochloremia, hyponatremia, and hypokalemia. He maintained a normotensive state, so laboratory results seemed less likely to be an adrenal etiology and more attributable to malnutrition. Consults for the Department of Social Services (DSS) and CPS were involved during the admission as well. The patient was placed into foster care and now remains in the care of his grandmother.

He was found to have intestinal malabsorption, compatible with being chronically malnourished and FTT. He was never specifically diagnosed with this, but his presentation was suggestive of something akin to Kwashiorkor, a severe protein malnutrition common in children in impoverished areas of the world. In addition to his FTT, the patient maintained persistent diarrhea, giving the diagnosis of chronic colitis. For the first year following his transplant, he had frequent loose stools and was found to have chronic Clostridioides difficile. To help clear this infection in the early stage of his immunodeficiency, the patient underwent a fecal microbiota transplant, which ultimately resolved the Clostridioides infection.

Following his PICU admission, in the setting of malnutrition, electrolyte abnormalities, and hepatosplenomegaly without jaundice or hyperbilirubinemia, a full gastrointestinal workup was pursued. He had repeat blood work completed approximately two weeks after his admission, which demonstrated the same results as those in the hospital (Table 8).

**Table 8:** CMP and liver enzymes following PICU admission CMP: Comprehensive metabolic panel, BUN: Blood urea nitrogen, GGT: Gamma-glutamyl transferase, ALT: Alanine transaminase, AST: Aspartate transaminase, ALP: Alkaline phosphatase

	Patient’s Value	Reference Value
Sodium	138	137 – 145 mmol/L
Potassium	3.5	4.0 – 5.3 mmol/L
Chloride	102	104 – 109 mmol/L
CO2	22	17 – 26 mmol/L
Anion Gap	14	7 – 15 mmol/L
Glucose	83	60 – 99 mg/dL
BUN	9.2	9.0 – 22.1 mg/dL
Creatinine	0.42	0.28 – 0.57 mg/dL
Bilirubin, total	0.3	0.0 – 0.4 mg/dL
GGT	276	0 -13 U/L
ALT	80	16 – 37 U/L
AST	140	25 – 47 U/L
ALP	841	143 – 318 U/L
BUN:Creatinine Ratio	21.9	7.3 – 21.7
Prealbumin	14	15 – 28 mg/dL
Albumin	4.5	4.2 – 5.0 g/dL

His liver enzymes were incidentally and persistently found to be elevated alongside the palpable hepatosplenomegaly. A liver biopsy revealed hepatitis but no autoimmune, cirrhosis, or fibrotic damage. He has had normal coagulation studies without right upper quadrant pain, jaundice, bloody stools, mouth sores, rashes, or joint pain, which makes a diagnosis of underlying liver disease unlikely. A GI scope was performed with concerns for gut GVHD. However, a definitive diagnosis of gut GVHD was indeterminate in the setting of his chronic GI infections. The pathology report noted acute inflammation in the sigmoid as well as features of chronic injury via scattered apoptotic bodies; there were primarily areas of 0-2 per 10 consecutive crypts and focal areas of 0-5 per 10 consecutive crypts. Other labs ordered to rule out differential diagnoses, such as antinuclear antibodies (ANA), ova and parasite (O+P), Helicobacter pylori, hepatitis panel, Cytomegalovirus (CMV), Epstein-Barr virus (EBV), stool elastase, and celiac panel, were unremarkable. He did have increased fecal fat, which is suggestive of malabsorption. The acute inflammation is likely attributable to intestinal inflammation in the setting of infectious diarrhea from Norovirus and Enteropathogenic Escherichia coli (EPEC). These infections affect his absorption abilities, and the malabsorption is represented clinically by his failure to gain weight and height. The EPEC infections showed resolution with azithromycin treatment; however, the Norovirus has been chronic. He takes a daily probiotic to aid with symptoms.

He was seen in the outpatient setting in August of this year (2024) for a hospital follow-up, where he was found to have chronic bronchiectasis, most prominent in the left lower lobe. He has had recurrent pneumonia infections unresponsive to antibiotics and a chronic baseline cough; bronchoscope culture revealed abundant growth of Hemophilus. He was brought in with his paternal grandmother, who received guardianship of him in September 2023. Per his grandmother’s report, this is the best the patient has been health-wise in a while. On exam, he was in no acute distress, interactive, thin, and of small frame. He had pallor, but no other dermatological abnormalities or lesions were appreciated. He had blepharitis of both eyes, left more prominent than right, and bilateral nasal congestion. His cardiac exam was unremarkable. On pulmonary auscultation, the patient had good air movement throughout all lung fields on the right and in the upper left lung fields: the lower left lung field had some diminished air movement, but otherwise, no rales, wheezes, or rubs were appreciated. He was treated with three weeks of Augmentin (USAntibiotics, Tennessee, US) with diminished cough quantity and resolution of sputum production. A preliminary workup to rule out cystic fibrosis was indeterminate via sweat chloride tests, and genetic testing is pending at the time this report was finalized. Cystic fibrosis, however, is unlikely in this patient because the basis of his recurrent infections is associated with his immunodeficient state following two bone marrow transplants.

## Discussion

The patient’s initial symptoms began with skin manifestations consistent with extramedullary hematopoiesis, which is sometimes referred to as “blueberry muffin” rash to describe its clinical feature. Due to the varying range of presentations of both benign and malignant nature, a full workup was initiated. The differential included intrauterine viral TORCH infections (Toxoplasmosis, Other (including syphilis, varicella, and parvovirus B19), Rubella, Cytomegalovirus, and Herpes simplex virus), hemolytic disease of the newborn, twin-to-twin transfusion syndrome, as well as neoplasms such as neuroblastoma and leukemias [8]. The evolving nature of the rash, both in size and in the areas affected, pointed toward a neoplastic process. Concerns for neuroblastoma were ruled out due to the lack of presence of abdominal masses or hepatosplenomegaly on physical exam and negative urine catecholamines, as shown in Table 9.

**Table 9:** Neuroblastoma workup VMA: vanillylmandelic acid, HVA: homovanillic acid

	Patient’s Value	Reference Value
VMA	12.1	< 25.0 mg/g
HVA	24.1	< 35.0 mg/g

Although the neuroblastoma workup was negative, a neoplastic process was still the primary consideration for diagnosis. The patient's gestational history was negative for confirmed multiparity, lack of maternal infections, or early jaundice. The skin biopsy findings pointed to aleukemic leukemia cutis, which is a rare condition in which leukemic cells invade only the skin, not involving the blood or bone marrow, much like this patient [9]. Aleukemic leukemia cutis should be differentiated from extramedullary hematopoiesis, as this finding on repeat skin biopsy presents as a rare precursor to leukemia, while extramedullary hematopoiesis may be a benign physiological process. This patient lacked leukocytosis at the time of both biopsies, as well as showed the absence of hepatosplenomegaly, which further negated the likelihood of extramedullary hematopoiesis. Extramedullary hematopoiesis results from blood cell formation outside the bone marrow, typically at the spleen, through compensatory measures. The patient was also found to have an orbital chloroma, which, similarly to aleukemic leukemia cutis, can be an early manifestation of myeloid neoplasms and be predictive of progression to more advanced disease. The presence of these physical exam findings encouraged further workup to develop a likely neoplastic diagnosis.

Histopathological evaluation is essential in initiating diagnosis; it helps not only to classify the MDS diagnosis but to further confirm the diagnosis as opposed to other bone marrow failure disorders such as aplastic anemia (AA). The distinction between MDS and AA is one of the largest diagnostic challenges in pediatric patients, especially in patients with low blasts [2]. Both AA and MDS have a resulting decrease in blood cell production, leading to similar clinical manifestations from varying degrees of pancytopenia, however, the pathogenesis differentiates them. AA is due to a failure of the bone marrow, while MDS is the presence of abnormally developed blood cells. Bone marrow biopsy of both AA and pediatric MDS tends to reveal hypocellular bone marrow. Hypocellular bone marrow may be more common in pediatric MDS patients as opposed to their adult counterparts. Another similarity between AA and MDS is the negative immunostaining for CD34. However, the presence of the left shift as well as dysplastic changes disqualifies AA as the primary diagnosis.

Since the etiology of aplastic anemia is broad - it can have a hereditary factor, as in the case of Fanconi anemia, and it can be linked to environmental exposures, it is plausible to also be considered in this patient. Although Fanconi anemia may be a precursor of early adulthood development of AML or MDS syndrome, this patient lacked the typical physical features of this condition. He did not have short stature preceding his bone marrow transplants, he lacked forearm and hand malformations, and his dermatological manifestations were more akin to petechiae than café-au-lait spots. Additionally, this patient lacks a familial history to point toward a hereditary bone marrow condition, and he was not exposed to excessive toxicities until after his MDS diagnosis. This is not to definitively exclude Fanconi anemia as an inciting agent to this patient’s early onset of MDS, as genetic testing was not done to determine the absence or presence of chromosomal breaks. Ultimately, at the time of this patient’s diagnosis, there was a lack of clinical evidence to point toward the sole diagnosis of Fanconi anemia, leading clinicians to pursue the more likely diagnosis of MDS.

The complex karyotype, in addition to abnormalities found in the bone marrow, does not suggest a specific disease entity; however, it consistently points toward a neoplastic myeloid process associated with underlying genetic instability. The differential diagnosis considered acute promyelocytic leukemia (APML); however, the FISH study lacked the typical 15:17 translocation, and APML was not consistent with the clinical features. Kostmann syndrome, a severe congenital neutropenia, was also considered but ruled out due to the absence of a varying degree of neutropenia. Kostmann syndrome tends to occur without physical manifestations other than the laboratory abnormality, making the appearance of this patient’s skin lesions unlikely. Even though aleukemic leukemia cutis and orbital chloroma tend to precede AML, repeated bone marrow findings continued to show a lack of systemic involvement of leukemic cells in the blood and bone marrow. This makes AML unlikely and helped lead to the diagnosis of MDS. This patient, like most other pediatric patients meeting the criteria for a MDS diagnosis, is placed in the refractory cytopenia category. He demonstrates the presence of 1% blasts with trilineage hematopoiesis and left-shifted myeloid cells. In patients with higher blast percentages, it is harder to differentiate between MDS and AML, further leaning away from an AML diagnosis [2].

The absence of ringed sideroblasts is important for pediatric MDS because it helps further eliminate possible diagnoses from the differential; it rules out sideroblastic anemia, drug toxicities, or nutritional deficiencies. Auer rods are pathognomonic for AML, so the absence of Auer rods makes MDS more likely than AML [1]. FISH analysis using a specific AML panel was unremarkable as well, further confirming AML as an unlikely diagnosis. Various studies have found abnormal karyotypes in 30%-50% of all children with MDS, and most of these are numerical anomalies of the chromosomes; fewer than 10% show structural abnormalities compared to karyotypes in patients with AML, where structural abnormalities predominate [2]. This is consistent with this patient’s findings, in which he predominantly had numerical anomalies. However, it is unique that he has the reassortment present between chromosomes 10 and 11. Combined with the other laboratory findings and clinical presentation, the diagnosis of MDS became more likely for this patient.

In this patient who did not have the typical demographic features for MDS, it was crucial to obtain all necessary diagnostics. Laboratory monitoring via CBC for cytopenia is a good starting point. Persistent deficiencies, both in the absence or presence of other somatic complaints, warrant further workup. This patient demonstrated physical manifestations in the absence of cytopenia until further progression of the disease process ultimately resulted in persistent anemia. Bone marrow biopsy ruled out more common bone marrow failure disorders, and karyotyping helped confirm an underlying neoplastic disease. The evidence of dysplastic changes, a left shift, and lack of systemic spread of leukemic cells were all key to filtering through the differential diagnosis.

The patient's BMT was found to be curative of MDS. However, the immunological response to the initial graft furthered his immunodeficient state and left him predisposed to delayed immunological recovery. Even though he was found to be in clinical remission, strict follow-up and clinical monitoring are of the utmost importance due to the associated morbidity as well as the potential for complications. This patient was lost to follow-up and eventually had to be admitted to the PICU for care. It is possible that some degree of his immunodeficient state from the transplant caused some of the resulting abnormalities, but it is also possible that there were home factors such as neglect.

Malnutrition, like restrictive eating disorders, such as anorexia nervosa, can present with electrolyte abnormalities and laboratory derangements, much like those of this patient at the time of admission to the PICU. It has been suggested that the mechanism of liver injury demonstrated by elevated AST and ALT is secondary to starvation, resulting in autophagy of liver cells [10]. However, there are hepatic presentations of GVHD that may present with an asymptomatic hypertransaminasemia [11]. A hepatic variant tends to demonstrate sharp elevations of ALT and AST with minimal increases in ALP in the setting of donor lymphocyte infusion or immunosuppression tapering but could also occur with an increase in ALP and GGT in the absence of jaundice. It is important to note that ALP levels tend to be more elevated in pediatric patients due to high bone turnover in the setting of growth, though high ALP in conjunction with elevated GGT points to a cholestatic disorder. The patient presents with significant elevations of AST, ALT, ALP, and GGT, which may be an uncommon mixed presentation of the above-mentioned hepatic variants of GVHD.

In developed countries, the leading cause of hypertransaminasemia is non-alcoholic fatty liver disease (NAFLD), which is highly unlikely in this patient due to his malnourished state as opposed to elevated BMI [12]. He does have a family history of diabetes in his mother, but his hemoglobin A1C has been evaluated and is well within normal limits. Another common cause is viral infections, such as hepatitis A, adenovirus, enterovirus, and likely norovirus, as is the case in the patient. This reiterates how challenging it is to differentiate acute viral infection from chronic GVHD. The presence of apoptotic bodies on biopsy points to GVHD: less than or equal to 2 apoptotic bodies per 10 crypts is typically normal. The patient demonstrated focal areas with a higher presence of apoptotic bodies, but the presence of acute inflammation may hinder the ability to make a diagnosis of exclusion. The inflammation could be attributable to multiple factors: chronic infection, GVHD, and malnutrition.

It is common for IgA levels to be transiently low during the first six months following BMT and rise back to normal levels one to two years after the transplant [13]. Children have lower IgA levels as compared to their adult counterparts in the same timeframes. Additionally, in patients who are also afflicted with GVHD, IgA levels will be lower than those without GVHD. This patient, unfortunately, has a persistent low IgA likely due to the additive effects of pediatric age and his chronic GVHD state. Regardless, this is a unique presentation of persistent deficiency. Selective IgA deficiency is the most common primary immunodeficiency, but this patient did not have symptoms of recurrent infections or recorded low IgA levels prior to his transplant, making the diagnosis of a primary deficiency unlikely. If the patient were solely IgA deficient, there may have been greater caution in utilizing IVIG because IVIG contains small amounts of IgA that may have resulted in an anaphylactic reaction to anti-IgA antibodies. However, being that he is deficient in both IgA and IgM, and the deficiencies occurred after the BMT, the benefits of receiving immunoglobulins were vast [14]. IVIG contains anti-inflammatory and immunomodulatory effects to help neutralize bacterial toxins and viruses, thus making it indicated in infection prevention after BMT. The transition to Hizentra as replacement therapy for his persistent hypogammaglobulinemia has helped to manage his deficiency as effectively as the IVIG infusions. Other pediatric patients with similar persistent deficiencies have been found to have comparable success, as well as a noted improvement in quality of life [15]. Hizentra is a subcutaneous injection that can be administered at home and provides similar benefits for this patient as those he was receiving with the IVIG infusions, while also giving him a less restricted approach to manage his hypogammaglobulinemia.

Although the patient has not officially obtained a diagnosis of GVHD, his clinical picture is concerning for the possibility. The chosen induction chemotherapeutics, as well as immunosuppressants, have been used in both the prevention and treatment of GVHD [6]. For example, cyclophosphamide demonstrates potent and selective activity against donor T cells, and mycophenolate mofetil has a cytostatic effect on lymphocytes. Other preventative agents used in this patient include rATG, rituximab, and maraviroc. Although his treatment was generated with the hope of decreasing GVHD in the future, his gastrointestinal symptoms could be attributed to gut GVHD. Symptoms of GVHD are not pathognomonic, making the diagnosis of GVHD a diagnosis of exclusion. Unfortunately, this patient has been afflicted with multiple gastrointestinal infections at different times, with the current setting of a chronic norovirus manifesting as persistent diarrhea as well as failure to thrive, making it hard to exclude viral etiology rather than GVHD. This failure to thrive may have an etiology attributable to his environmental neglect or malabsorption from persistent infections due to his hypogammaglobulinemia state.

In contrast to most patients diagnosed with MDS, this patient presented with normocytic anemia at the time of diagnosis rather than the typical macrocytic anemia. Macrocytic anemia tends to be refractory to both vitamin B12 and vitamin B9 replacements [1]. The refractory and persistent nature of the anemia in MDS patients can often allow enough time to pass for the MDS to progress to a leukemic state such as AML. This time to progression can be anywhere from months to years and is dependent on the severity of MDS as well as how early the diagnosis was made. Although ferritin tends to be low in the setting of iron deficiency anemia, the elevated ferritin may be connected to his malnourished state, as opposed to a more direct hematological pathology. This is concurrent with his elevated liver enzymes as well as elevated inflammatory markers (C-reactive protein (CRP) and erythrocyte sedimentation rate (ESR)), which may also be elevated as a manifestation of malnutrition. Additionally, prealbumin levels are a sensitive marker of nutritional status, more than albumin levels, due to the shorter half-life of prealbumin. About one week after his discharge, his prealbumin levels remained low, consistent with the picture of malnutrition.

This paints a complicated picture: it is hard to differentiate exactly how much his decreased immune competency is due to environmental neglect and how much is attributed to medical illness. His weakened immune state can be definitively linked to the bone marrow transplant, however, the significance of his complications makes it hard to discern exactly how much is attributable to either trait. It is possible some degree of his immunodeficiency is attributable to malnutrition, as well as poor follow-up leading to incomplete treatments and management of his complications.

Adverse childhood experiences (ACEs) such as parental neglect or abuse, familial dysfunction, exposure to illicit drugs or alcohol, or financial hardships can have a resulting impact on the health of a child [16]. Unfortunately, in the setting of a child with chronic health conditions, many of these characteristics are present: increased parental stress, diminished financial capabilities, and lack of family dynamics, thereby placing the chronically ill child at increased risk for neglect or abuse. There are physical manifestations of pain, such as abdominal pain or headache, as well as psychosocial results of anxiety, depression, and other mental health conditions, present in the child. Much like the interconnected complications the patient presented with, ACEs also have compounding effects: increased frequency of adverse exposures leads to an increased risk of poor future health outcomes. Adversity should be viewed as a factor affecting health rather than in isolation, considering the longstanding impacts of variables such as socioeconomic status and social determinants of health on young, at-risk individuals. Research is suggestive that a present, positive, and supportive role model serves as a protective factor in young children [16]. This accentuates the resilience of childhood: their ability to overcome the ACEs they have experienced and to continue to grow and develop. Since transitioning his guardianship to the care of his grandparents, the patient has exhibited the positive effects of a nurturing environment. He has been able to present to his specialist appointments and receive adequate treatment for the resolution of complicating manifestations with frequent follow-up care.

The chemotherapy alone would account for a one-to-two-year delay in bone age, rather than the significant delay he exhibited: his delay of almost three years suggests that other factors have contributed. BMT and TBI treatment have also been found to demonstrate GH deficiency in 82% of survivors with normal IGF-1 levels [17]. Although he is not GH deficient, he exhibited a lack of response or a dysfunctionality to the produced growth hormone. He was placed on Genotropin therapy, a recombinant growth hormone, to manage his growth failure. This treatment is more likely to be successful if it is started before puberty, prior to the closure of the epiphyseal plates, which is the case with this patient. This emphasizes the difficulty of attributing his varying clinical manifestations to medical or social factors.

## Conclusions

This patient presented with a unique constellation of manifestations because of his immunodeficient state in association with the rare occurrence of myelodysplastic syndrome in a pediatric patient at six months of age. Although myelodysplastic syndrome is predominantly found in older adults, this patient presented with only dermatological abnormalities that ultimately led to his myelodysplastic syndrome diagnosis. He lacked systemic involvement of atypical leukemic cells with minimal blasts, ruling out the more common acute myeloid leukemia diagnosis. Some diagnoses may be overlooked or not considered based on certain characteristics patients demonstrate, such as age or gender; however, this case signifies the importance of a thorough workup paired with a broad differential.

In addition to his uncommon diagnosis, his persistent immunodeficient state created a multitude of complications involving multiple organ systems. His subsequent care required the multidisciplinary approach of multiple medical specialties as well as social support from the Department of Social Services and Child Protective Services. His case demonstrates the importance for medical professionals to monitor for the possibility of all complicating factors, to have the skill set to manage medical conditions, and to be equipped to navigate social factors that play a role in determining health status.
